# Associations between birth characteristics and age-related cognitive impairment and dementia: A registry-based cohort study

**DOI:** 10.1371/journal.pmed.1002609

**Published:** 2018-07-18

**Authors:** Miriam A. Mosing, Cecilia Lundholm, Sven Cnattingius, Margaret Gatz, Nancy L. Pedersen

**Affiliations:** 1 Department of Medical Epidemiology and Biostatistics, Karolinska Institutet, Stockholm, Sweden; 2 Department of Neuroscience, Karolinska Institutet, Stockholm, Sweden; 3 Clinical Epidemiology Unit, Department of Medicine, Solna, Karolinska Institute, Stockholm, Sweden; 4 Center for Economic and Social Research, University of Southern California, Los Angeles, California, United States of America; 5 Department of Psychology, University of Southern California, Los Angeles, California, United States of America; University of Cambridge, UNITED KINGDOM

## Abstract

**Background:**

There is evidence for long-lasting effects of birth characteristics on cognitive ability in childhood and adulthood. Further, low cognitive ability throughout the lifetime has been linked to age-related cognitive decline and dementia risk. However, little is known about the effects of birth characteristics on cognitive dysfunction late in life. Here we explore potential associations between birth characteristics (weight, head circumference, length, and gestational age), adjusted and not adjusted for gestational age, and cognitive impairment and dementia late in life.

**Methods and findings:**

Data from twins in the Swedish Twin Registry born 1926–1960 were merged with information from the Swedish birth, patient, and cause of death registries, resulting in a sample of 35,191 individuals. A subsample of 4,000 twins aged 65 years and older also participated in a telephone cognitive screening in 1998–2002. Associations of birth characteristics with registry-based dementia diagnoses and on telephone-assessed cognitive impairment were investigated in the full sample and subsample, respectively. The full sample contained 907 (2.6%) individuals with a dementia diagnosis (an incidence rate of 5.9% per 100,000 person-years), 803 (2.4%) individuals born small for gestational age, and 929 (2.8%) individuals born with a small head for gestational age. The subsample contained 569 (14.2%) individuals with cognitive impairment. Low birth weight for gestational age and being born with a small head for gestational age were significant risk factors for cognitive dysfunction late in life, with an up to 2-fold risk increase (*p* < 0.001) compared to infants with normal growth and head size, even after controlling for familial factors, childhood socioeconomic status, and education in adulthood. In line with this, each additional 100 g birth weight and each additional millimeter head circumference significantly reduced the risk for dementia (hazard ratio 0.98, 95% confidence interval 0.97 to 0.99, *p* = 0.004) and cognitive impairment (odds ratio 0.99, 95% confidence interval 0.99 to 1.00, *p* = 0.004), respectively. Within-pair analyses of identical twins, though hampered by small sample size, suggested that the observed associations between birth characteristics and dementia are likely not due to underlying shared genetic or environmental etiology. A limitation of the present study is that registry-based dementia diagnoses likely miss some of the true dementia cases in the population. Further, a more precise measure of cognitive reserve early in life as well as a date of onset for the cognitive impairment measure in the subsample would have been favorable.

**Conclusions:**

In this study, we found that infants of smaller birth size (i.e., low birth weight or small head circumference adjusted and unadjusted for gestational age) have a significantly higher risk of age-related cognitive dysfunction compared to those with normal growth, highlighting the importance of closely monitoring the cognitive development of such infants and evaluating the potential of early life interventions targeted at enhancing cognitive reserve.

## Abstract 2

Using data from the Swedish Birth Registry, Miriam Mosing and colleagues investigate associations between healthy prenatal growth and cognitive dysfunction in late-life.

## Author summary

**Why was this study done?:**

- There is evidence for associations between birth characteristics and cognitive ability in childhood and adulthood.
- Low cognitive ability throughout the lifetime has been linked to age-related cognitive decline and dementia risk.
- Little is known about associations between birth characteristics and cognitive dysfunction late in life, such as dementia and cognitive impairment.

**What did the researchers do and find?:**

- We conducted a registry-based cohort study of 35,191 individuals linking data from the Swedish birth registry to the Swedish patient, cause of death, and twin registries to explore potential associations between birth characteristics and age-related cognitive dysfunction as measured by all-cause dementia and cognitive impairment.
- Low birth weight and small head circumference pose a significant risk for late life cognitive dysfunction.

**What do these findings mean?:**

- The findings highlight the importance of healthy prenatal growth and closely monitoring the cognitive development of small infants and those with signs of poor fetal growth.
- Potential interventions to increase cognitive reserve could be considered for infants with low birth weight and small head circumference.

## Introduction

Associations between cognitive function and anthropometric measures in childhood and adulthood, such as body height and head circumference, are well documented. Several studies have reported positive associations of cognitive ability with body length in childhood [1] and in adulthood [2,3], as well as with childhood and adult head circumference [2,4–6]. Further, both adult height and head circumference have been shown to be negatively associated with late life cognitive decline [6–8] and dementia [9–12].

Further, it has repeatedly been shown that adults with higher cognitive ability, higher education, and higher occupational achievements seem to be somewhat protected from developing age-related cognitive impairments such as dementia [13–16]. To account for this finding, the concept of cognitive reserve has been proposed, a concept that has gained much attention in regard to age-related cognitive decline [13]. The cognitive reserve theory proposes that differences in complex mental activity and ability throughout adulthood (cognitive reserve) as well as in brain structure and size (brain reserve) affect resilience against age-related decline [13,17]. This theory has been extended to childhood cognitive ability, with children scoring low on ability tests having an increased risk for dementia late in life [18]. Recently, a study found evidence that education not only has an enhancing effect on cognitive reserve but also contributes to brain reserve through increased cerebral volume [19]. These findings suggest that cognitive reserve is at least partly established early in life. Another possible explanation could be that other factors contribute to higher education and higher cognitive ability (i.e., cognitive reserve) as well as lower risk for age-related cognitive decline. Such factors could be influences very early in life, such as fetal growth patterns, gestational age at birth, or genetic influences.

Finally, birth characteristics, such as preterm birth, low birth weight, short birth length, small head circumference, and indicators of reduced fetal growth (i.e., small for gestational age), are independently associated with lower academic achievement, cognitive function, and intellectual performance in childhood [5,20–22] and young adulthood [23–25], even after controlling for familial factors and socioeconomic status. To the best of our knowledge, thus far only a few small studies [6,26,27] have explored associations between different birth characteristics and cognition in old age, reporting mixed findings. While some research reported significant associations for head circumference at birth, but not birth weight or length [26], other studies found no significant association between late life cognition or cognitive decline and the birth characteristics included, i.e., head circumference [6] or birth weight or length [27].

Together, the well-documented relationships between size at birth, gestational age, and cognition, as well as adult cognition and dementia, and the associations between adult anthropometric measures and dementia imply potentially adverse effects of reduced fetal growth for age-related cognitive decline and dementia. At this time, when population aging is one of the most important demographic phenomena in the world [28], and given the large burden of cognitive decline in older individuals [29,30], with currently no available cure for cognitive decline and dementia, research towards determining early predictors of cognitive impairment and dementia is warranted.

A link between birth characteristics and age-related cognitive dysfunction could in part explain the reported associations between adult anthropometric measurements, cognitive ability, and age-related cognitive dysfunction. Understanding these relationships is essential for the development of prevention strategies and to identify individuals who may benefit from early intervention. The present study explores potential associations between birth characteristics—i.e., weight, length, head circumference, and gestational age, as absolute measures as well as relative to gestational age—and all-cause dementia, using a large Swedish population-based study of more than 35,000 individuals. In addition, as registry-based dementia diagnoses may underestimate true numbers of cases of dementia in the population [31], associations between birth characteristics and age-related cognitive impairment based on comprehensive computer-assisted telephone screening were investigated in a subsample of 4,000 individuals.

## Methods

### Participants

The data were based on all twins registered with the Swedish Twin Registry (STR) [32,33] born between January 1, 1926, and December 31, 1960 (55 years and older at the end of follow-up on January 1, 2015), with information on birth characteristics from official birth records. Using the unique personal identification number assigned to all Swedish residents, individuals included in the STR with birth information were individually linked to the Swedish National Patient Register (NPR) and Cause of Death Register (CDR), resulting in a sample of 35,357 individuals. The NPR was introduced in Sweden in 1964 and contains information for all discharges from hospitals (with nationwide coverage since 1987), as well as hospital outpatient care since 2001, and includes dementia diagnoses (i.e., occurrence and date of first diagnosis) and vital status and other information. The CDR reached nationwide coverage in 1961 and contains information about underlying and contributory causes of death, including dementia diagnoses. Data from NPR and CDR were available until the end of 2014.

We excluded 55 individuals who had passed away before data from the NPR were available and 21 individuals with early onset dementia (onset before the age of 55 years) and 33 individuals because of Down syndrome, mental retardation, or cerebral palsy. An additional 57 individuals with impossible gestational age–birth weight combinations [34] were excluded from all analyses as their birth records may have been incorrect. The final dataset for the birth characteristics–dementia analyses contained 35,191 individuals.

Cognitive screening data from a telephone-based interview were available from all twins who were aged 65 years and older (*N* = 20,269) participating in the Screening Across the Lifespan Twin (SALT) study, conducted between 1998 and 2002 with all then living members of the STR [35]. A subsample of those with cognitive screening data (4,000 individuals) aged between 65 and 74 years (i.e., born 1926 to 1935) also had birth information available from official birth records and therefore formed part in the birth characteristics–cognitive impairment analyses. No birth information was available for those born prior to 1926. For further details on the cognitive screening data, see Gatz and colleagues [35].

This study was approved by the Regional Ethical Review Board in Stockholm (97:051, 2015/1729-31/5). Because this study was strictly register-based, individual informed consent was not deemed necessary [36]. Details on the application procedures for data usage are available on the homepages of the respective registries, i.e., the STR (http://ki.se/en/research/swedish-twin-registry-for-researchers), the NPR (http://www.socialstyrelsen.se/register/halsodataregister/patientregistret/inenglish), and the CDR (http://www.socialstyrelsen.se/statistics/statisticaldatabase/help/causeofdeath).

### Birth characteristics

Birth characteristics for the twins were derived from official birth records. Birth information was based on a nationwide collection (starting in 1926) of information from original birth records recorded by midwives and/or doctors at the time of birth. Birth record data were linked to collected data from the STR using the unique personal identification number assigned to all Swedish citizens. To minimize potential misclassification, only twins with known birth order were included. For twins born between 1926 and 1958, birth order was ascertained if the twins’ names could be found in the medical record or if they provided information on birth order in a later telephone interview. For twins born between 1959 and 1960, correct birth order was ascertained using an algorithm based on information from the medical record (birth weight, name if given at birth, and time of birth) in combination with information on birth weight and birth order from later data collections in the STR. For further details on the matching procedure see Hogberg and colleagues [37].

#### Birth weight (BW)

BW was explored as continuous raw weight scores (in grams divided by 100 for ease in interpreting statistical results) as well as dichotomized for low birth weight (LBW), coded as low (≤2,500 g) versus normal (>2,500 g). Further, 2 weight variables were derived adjusted for gestational age (GA) in days: (1) continuous birth weight adjusted for gestational age (BWGA) and sex, which was derived by regressing GA on BW and then standardizing the residuals by sex, and (2) small for gestational age (SGA), a statistical cutoff for the dynamic concept of poor fetal growth calculated here using the common Swedish definition of weighing less than 2 SD below the mean for a given GA and sex. Note that SGA is not synonymous with fetal growth restriction, where the fetus fails to reach its growth potential. Infants classified as SGA could be in this category for the following 3 reasons: (1) a non-malformed infant not having reached its growth potential (poor fetal growth), (2) a malformed infant for whom the fetal growth pattern is linked to malformations, and (3) a genetically small but otherwise normal infant. As the BW data nicely followed a normal distribution, and so as not to lose valuable information, extreme outliers more than 4 SD above/below the mean (7 in total) were Winsorized, i.e., set to 4 SD above or below the mean [38].

#### Head circumference (HC)

The potential predictive value of HC was explored as continuous raw variable (in millimeters), as well as adjusted for GA (in days) and sex (see above) as a continuous variable (head circumference adjusted for gestational age [HCGA]) and dichotomized for small for a given gestational age and sex (less than 2 SD below the mean) (small head circumference for gestational age [SHCGA]). As above, only 9 extreme outliers (more than 6 SD above/below the mean) were Winsorized [38].

#### Birth length (BL)

BL was similarly coded as a continuous raw variable (in centimeters), as well as adjusted for GA (in days) and sex (birth length adjusted for gestational age [BLGA]), and dichotomized for short for a given GA and sex (less than 2 SD below the mean) (short birth length for gestational age [SBLGA]). Six outliers (more than 5 SD below the mean) were Winsorized [38].

#### Gestational age (GA)

GA was coded as the number of weeks from the first day of the last menstrual period until birth. In addition, a dichotomous variable called preterm was derived, and individuals born before 37 gestational weeks were considered preterm.

### Measures of age-related cognitive dysfunction

#### Dementia

Register-based dementia diagnoses (coded as a dichotomous variable) as well as date of first diagnosis were derived from the NPR and CDR, including all primary and contributory diagnoses in the NPR and all underlying and contributory causes of death in the CDR. The various ICD codes used to detect the different types of dementia (Alzheimer disease, vascular dementia, and other dementia) are shown in S1 Table.

#### Cognitive impairment

Computer-assisted telephone cognitive screening was done in 2 stages. First, participants were interviewed with an instrument known as TELE [39,40]. The TELE includes questions about health and daily functioning, a 10-item mental status questionnaire (MSQ) [41], 3-word recall, serial 3s, and a word similarities task (3 pairs). Participants received a score on the TELE ranging from 0 to 19. Second, if participants performed poorly on the TELE (<13.5), an informant was interviewed using the Blessed Dementia Rating Scale (BDRS) [42] to find out how much the participant’s cognitive status interfered with daily functioning. Participants were considered cognitively impaired if they performed poorly in 2 or more domains of the TELE, needed help with activities of daily living due to memory problems, had a BDRS score of at least 1.5 (based on the established cutoff for functional impairment [43]), or made more than 2 errors on the MSQ questions [44]. In the present study, to create a dichotomous variable, other participants were considered cognitively intact. Approximately half of those considered cognitively impaired based on telephone screening were subsequently clinically diagnosed with dementia, and another 13% with mild cognitive impairment [35].

### Covariates

In addition to sex and age/year of birth (YOB; coded as a 10-year interval) of the participants, a number of other factors that could potentially also influence birth characteristics and age-related cognitive dysfunction were included as covariates where applicable. For descriptive information on covariates see S2 Table.

#### Prenatal covariates

Parity (the number of children previously born to the same mother) and age of mother at birth were included as covariates where applicable.

#### Socioeconomic status (SES) at birth

Birth SES is a well-known predictor of child cognitive development, with lower SES being associated with lower IQ [45,46], and could impact education and as such cognitive reserve later in life. In addition, lower SES has been shown to be associated with a slightly increased risk of adverse birth characteristics [47]. Birth SES was based on the father’s occupation (or mother’s occupation if the father’s occupation was missing) as documented in midwife records, coded into the following 3 classes: higher class—large business owners/senior officials; middle class—lower level, small business owners; and worker class—workers in private or public services. This categorization for social class was created in 1911 and was commonly used until the 1970s in Sweden [48].

#### Education level

Education level of the participants was also included as it has been shown to be a strong marker of cognitive reserve [19]. Education was regarded as low if individuals had less than 9 years of education and as high if they had 9 or more years of education.

### Multiple imputation

As some of the variables (see below) had missing values, resulting in reduced *N*s in those models corrected for increasing numbers of covariates, the multiple imputation (MI) procedure in StataIC 14 [49] was used to impute missing data. MI is a simulation-based approach that uses all known variables in the model to replace missing values with multiple sets of simulated values, rather than a single value, to complete the data [50]. That way, MI preserves the distributions and relationships in the data while still reflecting the uncertainty in the predictions of the missing values. All variables forming part of our analyses were also included in our imputation models (including the outcome and fully observed variables). The variables with missing data included in the imputation model were HC (continuous), BL (continuous), BWGA (continuous or dichotomous, i.e., SGA), HCGA (continuous or dichotomous), BLGA (continuous or dichotomous), GA (continuous or dichotomous), parity (continuous), age of mother (continuous), birth SES (ordinal), and education level (dichotomous). In addition, the fully observed variables BW, YOB, sex, age, zygosity, LBW, and either dementia diagnosis and the cumulative hazard function or cognitive impairment were also included to preserve their relationships with the imputed variables. Note that imputations for each model were run twice, first with the continuous GA variables (i.e., BWGA, HCGA, BLGA, and GA) and then a second time with the dichotomized GA variables (i.e., SGA, SHCGA, SBLGA, and preterm). Further, mother’s age only had missing values in the dementia analyses while it was included as a fully observed variable for the cognitive impairment analyses. To calculate how many imputations should be regarded as sufficient for the pattern of missing data in the 2 samples, a 2-step procedure using a quadratic rule recommended by von Hippel [51] was used. Accordingly, 58 and 100 imputed datasets were created in each run for the dementia dataset and the cognitive impairment subset, respectively, using chained equations (using the STATA command *mi impute chained*), a MI method using a sequence of univariate imputations with fully conditional specification of prediction equations [49]. The augmented regression option was used as there was perfect prediction. The resulting estimates were combined using Rubin’s rule to derive 1 final MI result. See Table 1 for the distribution of missing/imputed values for each variable and set of analyses, i.e., dementia and cognitive impairment. The subsequent analyses (described below) were conducted using the imputed datasets. For comparison and to check whether the imputation process altered our findings, all analyses were then repeated in the original dataset with missing values and in a reduced dataset including only individuals with complete data. The results of these additional analyses are reported in S3–S6 Tables.

**Table 1:** Patterns of missingness in the birth variables and covariates before imputation.

Variable	Birth characteristics–dementia, *N* = 35,191		Birth characteristics–cognitive impairment, *N* = 4,000	
	Complete	Missing/imputed	Complete	Missing/imputed
BW (in 100 g)/LBW	35,191	0	4,000	0
BWGA/SGA	33,707	1,484	3,702	298
HC (in mm)	34,166	1,025	3,721	279
HCGA/SHCGA	32,754	2,437	3,449	551
BL (in cm)	34,979	212	3,931	69
BLGA/SBLGA	33,521	1,670	3,646	354
Parity	34,816	375	3,927	73
Mother’s age	35,174	17	4,000	0
Gestational age/preterm	33,707	1,484	3,702	298
Birth year	35,191	0	4,000	0
Education level	28,934	6,257	3,879	121
Birth SES	25,505	9,686	1,781	2,219

BL, birth length; BW, birth weight; BLGA, birth length adjusted for gestational age; BWGA, birth weight adjusted for gestational age; HC, head circumference; HCGA, head circumference adjusted for gestational age; LBW, low birth weight; SES, socioeconomic status; SGA, small for gestational age; SHCGA, small head circumference for gestational age; SBLGA, small birth length for gestational age.

### Statistical analyses

Though there was no specific study protocol for the present study, all analyses were carefully planned with a statistician and decided on before the analyses were conducted. The only data-driven change to the analyses was the MI process, conducted to account for missingness in some of the covariates. While originally only 10 and then 40 datasets were imputed, this was increased to 58 and 100 for the full sample and subsample, respectively, following recommendations by von Hippel [51] and Graham et al. [52] on how many datasets to impute, after one reviewer’s comments led us to investigate this matter further.

### Birth characteristics and dementia

Survival analyses were conducted to explore the association between birth characteristics (BW [BW raw continuous score, LBW, BWGA, and SGA], HC [HC raw continuous, HCGA, and SHCGA], BL [BL raw continuous, BLGA, and SBLGA], and GA [GA continuous in weeks and preterm]) and dementia. The number of months of survival (age) from date of birth to date of dementia diagnosis or date of censoring (death or end of follow-up on January 1, 2015) was used as the time scale (information on vital status was drawn from the CDR and linked to the present dataset). Cox proportional hazards regression analysis was used to calculate hazard ratios (HRs). HRs represent the effect of a 1-unit change in the predictor (e.g., BW) on the base dementia risk (raw hazard of dementia) during the follow-up period. Study participants were followed from 1977 (when data from the NPR were available). To correct for correlation within twin pairs, the robust standard error estimator for clustered observations was used. Four models were fitted for each of the birth characteristics: (1) without any covariates; (2) corrected for sex, YOB, parity, and age of mother; (3) corrected for birth SES in addition to the covariates included in model 2; and (4) corrected for education level in addition to all covariates included in model 3. The assumption of proportional hazards was tested for each model using Schoenfeld residuals [53]. No evidence for deviation from the proportional hazards assumption was found.

### Birth characteristics and cognitive impairment

To obtain odds ratios (ORs), logistic regressions were conducted with the dichotomized cognitive impairment score as outcome variable and BW (BW raw continuous score, LBW, BWGA, and SGA), HC (HC raw continuous, HCGA, and SHCGA), BL (BL raw continuous, BLGA, and SBLGA), and GA (continuous in weeks and preterm) as the predictors. Again, robust estimators of standard errors for clustered data were used. Initially, a baseline model was fitted for each of the birth characteristics and the outcome variable separately without correcting for any of the covariates. The analyses were then repeated including the various covariates, i.e., age (at cognitive screening), sex, parity, and age of mother, birth SES, and education level (see above description of the 4 models).

### Within-pair analyses in identical twins

To further explore associations with regard to familial (genetic and environmental) factors shared within the twin pairs, within-pair (co-twin control) analyses in identical pairs were conducted. Identical (i.e, monozygotic [MZ]) twins share all their segregating genes as well as their family environment. If adverse birth effects cause a risk for cognitive dysfunction, we would expect that a twin with more unfavorable birth characteristics would have a higher risk for cognitive dysfunction than his or her co-twin with more favorable birth characteristics (e.g., higher BW). Note that only complete identical twin pairs discordant for exposure (i.e., with a difference in birth characteristics) and outcome (i.e., cognitive impairment/age at dementia diagnosis) contributed to the within-pair analyses. Conditional (Cox) regression models with family identification as the stratum variable were conducted for dementia, thereby fixing individual baseline hazard within pairs while allowing it to vary between pairs [54]. In a matched sample like this, the conditional logistic regression estimates the effect of the difference between the 2 observations in the strata. Thus, a continuous measure of, for example, BW estimates a potential linear effect of the within-pair difference in BW. As such, twins can be regarded as discordant for the exposure if they differ in the respective birth characteristic even by just 1 unit, and for the outcome if they do not get dementia simultaneously. Similarly, conditional logistic regression was conducted for identical twin pairs discordant on cognitive impairment. Note that the within-pair analyses did not require correction for covariates as each twin was matched to his or her co-twin who shared the same age, sex, parity, age of mother at birth, and GA (in addition to many other unmeasured environmental influences), and therefore missing covariates were not an issue in these analyses. Hence, within-pair analyses were conducted only in the unimputed dataset. Also, note that these analyses were corrected for GA, although the exposure variables as such were unadjusted. To increase power, analyses were repeated including also same-sex dizygotic (DZ) twin pairs as well as an interaction effect for zygosity and exposure to test whether the effect of the birth characteristic differed between MZ and DZ twins. All analyses were conducted in StataIC 14 [49].

### Additional analyses with birth order

One of reviewer’s comments led us to investigate further whether birth order within pairs may play a role in late life cognitive dysfunction. The second-born twin is frequently the smaller one and also has a higher risk for asphyxia, which may result in lower cognitive ability and could therefore mediate potential associations between birth characteristics and cognitive dysfunction late in life. We therefore tested whether birth order (corrected for sex) had a main effect on the 2 cognitive outcomes and then repeated the imputation process and all main analyses, including the co-twin control analyses, adjusting for birth order.

## Results

### Descriptive statistics

The final dataset for the birth characteristics–dementia analyses contained 35,191 twin individuals aged 55 to 89 years (mean = 69.21, SD = 8.83) at follow-up, with information on at least 1 of the birth characteristics and dementia diagnosis (Table 2). BW ranged between 1,010 and 4,700 g (mean = 2,664), HC ranged between 230 and 430 mm (mean = 329.5), and BL ranged between 34 and 59 cm (mean = 47.7)—expected birth characteristics in a twin sample [55]. Of this sample, 2.6% were diagnosed with dementia (an incidence rate of 5.9% per 100,000 person-years), with a mean age at diagnosis of 74.31 years (SD = 7.38, range = 55–88). Of the 4,000 individuals in the subsample included in the cognitive impairment analyses (mean age at screening = 68.36 years, SD = 2.55, range = 65–74), 14.2% were identified as cognitively impaired based on the telephone screening. Descriptive statistics for the birth characteristics and cognitive variables in both samples are presented for sexes separately and combined in Table 2. Additional testing for a main effect of birth order on the 2 measures of cognitive dysfunction showed that although the second-born twin was on average somewhat smaller than the first-born, birth order was far from significant (*p* > 0.5) for dementia and cognitive impairment, suggesting no association between birth order and cognitive dysfunction.

**Table 2:** Descriptive statistics for the birth and cognitive variables (unimputed).

Variable	Dementia sample			Cognitive impairment sample		
	All	Males	Females	All	Males	Females
***N***	34,166–35,191	16,324–16,813	17,842–18,378	3,727–4,000	1,744–1,876	1,983–2,124
**BW (100 g)**	2,664 (505.7)	2,727 (506.8)	2,607 (497.9)	2,678 (508.5)	2,747 (505.6)	2,618 (503.5)
**LBW**	12,752 (36.2%)	5,335 (31.7%)	7,417 (40.4%)	1,566 (39.2%)	629 (33.5%)	938 (44.2%)
SGA*	801 (2.4%)	372 (2.3%)	429 (2.4%)	83 (2.7%)	49 (2.8%)	51 (2.6%)
**HC (mm)**	329.5 (17.5)	332.6 (17.1)	326.7 (17.4)	332.3 (16.8)	335.7 (16.2)	329.3 (16.8)
SHCGA*	920 (2.8%)	420 (2.7%)	500 (2.9%)	107 (3.1%)	51 (3.1%)	56 (3.1%)
**BL (cm)**	47.7 (2.8)	48.1 (2.7)	47.3 (2.8)	47.9 (2.7)	48.3 (2.7)	47.5 (2.7)
SBLGA*	988 (2.9%)	454 (2.8%)	530 (3.0%)	105 (2.9%)	40 (2.3%)	62 (3.2%)
**GA (weeks)**	37.8 (2.5)	37.7 (2.5)	37.8 (2.5)	37.8 (2.4)	37.8 (2.5)	37.9 (2.4)
**Preterm**	9,559 (28.4%)	4,661 (29.0%)	4,898 (27.8%)	1,020 (27.6%)	481 (27.6%)	539 (27.5%)
**Dementia**	907 (2.6%)	415 (2.5%)	492 (2.7%)	471 (11.8%)	209 (11.1%)	262 (12.3%)
Age at diagnosis (years)	74.3 (2.6)	73.8 (7.4)	74.8 (7.4)	78.7 (5.0)	78.4 (5.2)	79.0 (4.9)
**Cognitive impairment**	—	—	—	569 (14.2%)	270 (14.4%)	299 (14.1%)
Age at screening (years)	—	—	—	68.4 (2.6)	68.4 (2.5)	68.3 (2.6)

Data are given as mean (SD) or count (percent).

*As GA was not available for everyone, the *N*s for the GA-corrected variables are somewhat reduced in the total sample (*N* = 32,754–33,702) and in the subsample (*N* = 3,454–3,702).

BL, birth length; BW, birth weight; GA, gestational age; HC, head circumference; LBW, low birth weight; SBLGA, short birth length for gestational age; SGA, small for gestational age; SHCGA, small head circumference for gestational age.

### Birth characteristics–dementia analyses

Table 3 shows the associations between indices of BW, HC, BL, and GA and dementia diagnosis from analyses both not adjusted and subsequently adjusted for covariates based on the imputed dataset. Regardless of the covariates included, lower BW significantly increased the risk for dementia, with an approximately 2% reduction in dementia risk with each additional 100 g BW and a 19%–23% increase of dementia risk for infants with LBW. Similarly, each additional standard deviation BWGA resulted in an 8%–9% significantly reduced risk for dementia, and SGA infants showed a 19% higher dementia risk, though the latter association was not significant.

**Table 3:** Hazard ratios for dementia diagnosis based on survival analyses in relation to birth characteristics.

Variable	Model 1		Model 2		Model 3		Model 4	
	HR (95% CI)	*p*-Value	HR (95% CI)	*p*-Value	HR (95% CI)	*p*-Value	HR (95% CI)	*p*-Value
BW (100 g)	**0.98 (0.97–1.00)**^#^	**0.016**	**0.98 (0.97–0.99)**	**0.004**	**0.98 (0.97–0.99)**	**0.004**	**0.98 (0.97–0.99)**	**0.004**
LBW	**1.19 (1.04–1.36)**	**0.011**	**1.23 (1.07–1.41)**	**0.003**	**1.23 (1.07–1.41)**	**0.004**	**1.22 (1.07–1.40)**	**0.004**
BWGA	**0.92 (0.86–0.99)**	**0.017**	**0.91 (0.85–0.98)**	**0.008**	**0.91 (0.85–0.98)**	**0.008**	**0.91 (0.85–0.98)**	**0.008**
SGA	1.19 (0.81–1.76)	0.380	1.19 (0.81–1.77)	0.374	1.19 (0.81–1.76)	0.381	1.19 (0.80–1.76)	0.420
HC (mm)	1.00 (0.99–1.00)	0.304	1.00 (0.99–1.00)	0.209	1.00 (0.99–1.00)	0.218	1.00 (0.99–1.00)	0.220
HCGA	0.97 (0.90–1.04)	0.401	0.97 (0.90–1.04)	0.402	0.97 (0.90–1.04)	0.413	0.97 (0.90–1.04)	0.415
SHCGA	**1.67 (1.15–2.41)**	**0.007**	**1.65 (1.14–2.39)**	**0.008**	**1.65 (1.14–2.39)**	**0.008**	**1.65 (1.14–2.39)**	**0.008**
BL (cm)	0.98 (0.96–1.01)	0.136	0.98 (0.95–1.00)	0.071	0.98 (0.95–1.00)	0.073	0.98 (0.95–1.00)	0.073
BLGA	0.96 (0.89–1.02)	0.195	0.95 (0.89–1.02)	0.166	0.95 (0.89–1.02)	0.166	0.95 (0.89–1.02)	0.167
SBLGA	1.40 (0.99–1.98)	0.058	1.40 (0.98–1.98)	0.063	1.40 (0.98–1.98)	0.062	1.40 (0.98–1.98)	0.062
GA (weeks)	0.99 (0.96–1.02)	0.461	0.99 (0.96–1.02)	0.384	0.99 (0.96–1.02)	0.392	0.99 (0.96–1.02)	0.396
Preterm	0.96 (0.82–1.13)	0.652	0.97 (0.83–1.14)	0.733	0.97 (0.83–1.14)	0.730	0.97 (0.82–1.14)	0.729

Estimates are shown unadjusted (model 1) and adjusted for YOB (in 10-year intervals), sex, age of mother, and parity (model 2); YOB, sex, age of mother, parity, and birth SES (model 3); and YOB, sex, age of mother, parity, birth SES, and education level (model 4). Missing variables were imputed (*N* = 35,191 for all dementia analyses). Significant estimates are in bold.

^#^Upper CIs of 1.00 for significant estimates are rounded (i.e., they are below 1.000 but higher than 0.995).

BL, birth length; BLGA, birth length adjusted for gestational age; BW, birth weight; BWGA, birth weight adjusted for gestational age; GA, gestational age; HC, head circumference; HCGA, head circumference adjusted for gestational age; HR, hazard ratio; LBW, low birth weight; SBLGA, small birth length for gestational age; SES, socioeconomic status; SGA, small for gestational age; SHCGA, small head circumference for gestational age; YOB, year of birth.

While there was not sufficient evidence in support of an association between the 2 continuous HC variables, i.e., HC (in mm) and HCGA, and dementia, infants with SHCGA had an about 65% higher risk for dementia, independent of the covariates included. Though the estimates were in the expected direction for all 3 BL variables, with an up to 40% increased risk for dementia for those born with SBLGA, none of the estimates were significant. Similarly, there was little evidence in support of an association between GA or preterm and dementia. Of the covariates, parity and birth decade were the only significant risk factors for dementia, with increased parity (*p* < 0.05) and later birth decade (with the exception of the most recent birth decade, 1946 to 1955) somewhat increasing the risk for dementia, i.e., those born between 1936 and 1945 showed a somewhat increased risk (non-significant), while those born between 1946 and 1955 had an about 40% higher risk compared to those born 1926 to 1935 (*p* < 0.05). There was little evidence in support of an association between birth SES or education and dementia (even when included without any of the birth-related variables). Note that overall results based on the full unimputed sample as well as the reduced sample (only participants with all covariates) were very similar in terms of both effect sizes and significance levels, although the latter varied slightly between analyses depending on the sample size (see S3 and S5 Tables). Further, adjustment for birth order had no effect on any of the results (see S7 Table).

### Birth characteristics–cognitive impairment analyses

Table 4 shows associations between indices of BW, HC, BL, and GA and cognitive impairment from analyses both uncorrected and subsequently corrected for covariates in the imputed subsample. While there was little evidence in support of an association between BW, LBW, or BWGA and cognitive impairment, ORs for SGA ranged between 1.69 and 1.76 depending on the covariates included, suggesting a somewhat increased risk of cognitive impairment for infants born SGA.

**Table 4:** Odds ratios for cognitive impairment based on logistic regression in the subsample in relation to birth characteristics.

Variable	Model 1		Model 2		Model 3		Model 4	
	OR (95% CI)	*p*-Value	OR (95% CI)	*p*-Value	OR (95% CI)	*p*-Value	OR (95% CI)	*p*-Value
BW (100 g)	1.00 (0.98–1.02)	0.702	0.99 (0.97–1.01)	0.558	1.00 (0.98–1.02)	0.685	1.00 (0.98–1.02)	0.717
LBW	0.93 (0.77–1.12)	0.423	1.00 (0.82–1.20)	0.962	0.98 (0.81–1.19)	0.846	0.97 (0.81–1.18)	0.801
BWGA	0.98 (0.89–1.08)	0.698	0.94 (0.85–1.04)	0.243	0.95 (0.86–1.05)	0.320	0.95 (0.86–1.05)	0.316
SGA	**1.69 (1.01–2.83)**	**0.045**	**1.76 (1.04–2.97)**	**0.035**	**1.71 (1.00–2.90)**	**0.048**	**1.73 (1.00–2.99)**	**0.048**
HC (mm)	**0.99 (0.99–1.00)**^#^	**0.036**	**0.99 (0.99–1.00)**^#^	**0.005**	**0.99 (0.99–1.00)**^#^	**0.007**	**0.99 (0.98–1.00)**^#^	**0.009**
HCGA	**0.86 (0.79–0.95)**	**0.003**	**0.84 (0.77–0.93)**	**0.001**	**0.85 (0.77–0.94)**	**0.001**	**0.85 (0.77–0.94)**	**0.001**
SHCGA	**2.15 (1.39–3.33)**	**0.001**	**2.26 (1.47–3.46)**	**<0.001**	**2.23 (1.45–3.43)**	**<0.001**	**2.24 (1.45–3.46)**	**0.000**
BL (cm)	1.00 (0.96–1.03)	0.917	0.99 (0.95–1.02)	0.449	0.99 (0.95–1.02)	0.504	0.99 (0.95–1.03)	0.534
BLGA	0.96 (0.87–1.05)	0.353	0.94 (0.85–1.03)	0.174	0.94 (0.85–1.03)	0.198	0.94 (0.85–1.03)	0.196
SBLGA	1.51 (0.92–2.48)	0.101	1.55 (0.94–2.54)	0.085	1.56 (0.95–2.57)	0.080	1.61 (0.97–2.66)	0.064
GA (weeks)	1.03 (0.99–1.07)	0.129	1.02 (0.98–1.06)	0.297	1.02 (0.98–1.06)	0.276	1.02 (0.98–1.07)	0.233
Preterm	0.86 (0.70–1.07)	0.175	0.91 (0.73–1.13)	0.397	0.91 (0.73–1.13)	0.387	0.90 (0.73–1.12)	0.359

Estimates are shown unadjusted (model 1) and adjusted for age, sex, age of mother, and parity (model 2); age, sex, age of mother, parity, and birth SES (model 3); and age, sex, age of mother, parity, birth SES, and education level (model 4). Missing variables were imputed (*N* = 4,000 for all cognitive impairment analyses). Significant estimates are in bold.

^#^Upper CIs of 1.00 for significant estimates are rounded (i.e., they are below 1.000 but higher than 0.995).

BL, birth length; BLGA, birth length adjusted for gestational age; BW, birth weight; BWGA, birth weight adjusted for gestational age; GA, gestational age; HC, head circumference; HCGA, head circumference adjusted for gestational age; LBW, low birth weight; SBLGA, small birth length for gestational age; SES, socioeconomic status; SGA, small for gestational age; SHCGA, small head circumference for gestational age.

All 3 measures of HC had a significant influence on cognitive impairment regardless of the covariates included, with a 1% reduction in odds with each additional millimeter HC, about 15% lower odds for each additional standard deviation HCGA, and a more than 2-fold greater odds for individuals with SHCGA at birth. Which covariates were included had little influence on effect sizes and significance levels. Again, there was little evidence in support of an association between any BL or GA variables and cognitive impairment, although infants born with SBLGA showed a 51%–61% increased odds for cognitive impairment (non-significant). Of the covariates, age, parity, and education were significant, with 7% higher odds of being diagnosed with cognitive impairment with each additional year of age (*p* < 0.01) and parity number (*p* < 0.01), as well as a 35% lower odds for those with higher education (*p* < 0.001). Again, results based on the full unimputed sample as well as the reduced sample (only participants with all covariates) were very similar in terms of both effect sizes and significance levels, although the latter varied slightly between analyses depending on the sample size (see S4 and S6 Tables). Additional adjustment for birth order had no effect on any of the results (see S8 Table).

### Within-pair analyses

Within-pair analyses in twins were performed for BW and HC (both converted to *z*-scores to compare results with the between-pair analyses of the BWGA and HCGA variables, which also were standardized) to explore whether the significant associations observed in the above analyses were confounded by underlying shared liability (Table 5). In total, there were 182 MZ pairs who were discordant for dementia diagnosis (days between dementia diagnosis of 1 twin and diagnosis or censoring of the co-twin: mean = 2,215, SD = 2281, range = 32–13,364) and 62 who were discordant for cognitive impairment. HRs and ORs in the within-pair analyses showed a similar effect of birth characteristics compared to the GA-adjusted results for BW and HC from the between-pair analyses (i.e., BWGA and HCGA), though they were not significant. When including both MZ and DZ twins in the analyses, effect sizes were still not significantly different from 1, and also the interaction effect was non-significant, suggesting that there was no significant difference in effect size between MZ and DZ twins. Adjusting for birth order did not change results significantly (see S9 Table). However, while effect sizes remained comparable for cognitive impairment, the point estimates for dementia risk changed somewhat, with wide confidence intervals reflecting reduced power. Considering the risk for misclassification in registry data (the source of the dementia diagnoses) and that such misclassification has a bigger impact in co-twin control analyses as compared to cohort analyses [56], it is unlikely that the change in effect size reflects an effect of birth order on the associations with dementia.

**Table 5:** Results of within-pair analyses in twin pairs discordant for birth characteristics and dementia or cognitive impairment, adjusted for familial factors shared within twin pairs.

Variable	MZ pairs only			MZ and same-sex DZ pairs		
	*N* pairs	HR or OR (95% CI)	*p*-Value	*N* pairs	HR or OR (95% CI)	*p*-Value
**Dementia**						
Birth weight (*z*-score)	174	0.81 (0.53–1.24)	0.334	461	0.90 (0.71–1.15)	0.399
Head circumference (*z*-score)	131	0.83 (0.53–1.29)	0.403	340	0.92 (0.71–1.21)	0.562
**Cognitive impairment**						
Birth weight (*z*-score)	54	0.76 (0.39–1.49)	0.497	161	0.92 (0.64–1.31)	0.645
Head circumference (*z*-score)	40	0.77 (0.36–1.64)	0.422	118	0.88 (0.58–1.34)	0.546

DZ, dizygotic; HR, hazard ratio; MZ, monozygotic; OR, odds ratio.

## Discussion

The present study explored associations between birth characteristics (BW, BL, HC, and GA) and dementia and cognitive impairment later in life. Overall, lower BW and smaller HC showed associations in the expected direction, resulting in increased risks for age-related cognitive dysfunction, i.e., dementia or cognitive impairment. For dementia, lower BW (independent of GA) was a significant risk factor, with a 2% higher risk with each 100 g lower BW and about 22% higher risk if weighing less than 2.5 kg at birth. Similar associations were observed when BW was adjusted for GA, with a 9% risk reduction for dementia for each additional standard deviation BWGA and a 17% risk increase for individuals born SGA, though the latter was not significant. HC was a significant risk factor only when the head was SGA (65% risk increase), suggesting harmful effects of reduced fetal growth for dementia. Similarly, detrimental effects were observed for cognitive impairment, with individuals born SGA and with SHCGA showing approximately 73% and 124% higher odds, respectively. In addition, larger HC was, independent of GA as well as adjusted for GA, a significant protective factor for cognitive impairment (but not for dementia), with each additional millimeter resulting in a 1% lower odds for cognitive impairment and each additional SD HCGA resulting in a 15% lower odds. BL and GA (independent of other birth characteristics) showed no significant association with late life cognitive dysfunction. Overall, these findings suggest negative long-term effects of small birth size on risk of cognitive dysfunction late in life, with additional protective effects of each extra 100 g of BW for dementia and each additional millimeter of HC for cognitive impairment (independent of GA).

Past research has shown birth characteristics adjusted and unadjusted for GA to be independently associated with academic achievement, cognitive function, and intellectual performance early in life [5,20–25], traits known to be protective against cognitive dysfunction late in life and frequently referred to as cognitive reserve [14]. The exact mechanisms underlying such long-term effects of birth characteristics are unclear, although some research has suggested that fetal growth restriction can result in structural brain differences, such as reduced grey matter [57,58], i.e., lower brain reserve. In view of the previous literature, the present findings suggest that the reported protective value of cognitive reserve (including brain reserve) against cognitive dysfunction may at least to some degree be explained by factors influencing fetal growth and development.

Contrary to prior literature and the findings of another study using a partly overlapping sample in the STR [59,60], education level had little impact on dementia diagnoses (even when included as the sole predictor). The same was observed for birth SES. There are several possible explanations for the missing protective effect of these potential indicators of early and later cognitive stimulation on dementia. First, in the present sample only individuals who had birth information could be included, restricting the maximum age to 89 years. As a result, we may have an overrepresentation of individuals diagnosed towards the young end of the typical age of onset of dementia, and, as one effect of cognitive reserve is to delay dementia, we may miss those protected by cognitive reserve. Second, registry-based dementia diagnoses have been shown to capture only about 63% of the true number of dementia cases in the population, despite showing a near perfect specificity [31,61]. Consequently, individuals may have been misclassified as healthy, resulting in a bias towards null. In that case, it is even more remarkable that we still observe such strong associations between birth characteristics and dementia, suggesting that such associations may be stronger than birth SES, education, and possibly cognitive reserve. Third, it could be that individuals with higher education and higher SES are more likely to seek medical advice when they or their family members notice cognitive symptoms and, as such, are more likely to be diagnosed with dementia. This would result in lower sensitivity of dementia diagnosis among lower educated individuals, which in turn could cover up the protective effect of education. Further, though the effect of education on dementia has been shown to be quite consistent [62], previous research has suggested that it varies somewhat depending on the study population [30,62] and is most consistent when years of education reflect cognitive capacity [63,64]. Therefore, another possible explanation could be that the 2 measures used here do not very well reflect true cognitive capacity and as such do not affect cognitive reserve, especially given that the education measure included was coarse, with only 2 categories. However, a significant association of education, but not childhood SES, was observed with the cognitive impairment measure. The association between education and cognitive impairment was mostly independent from the birth characteristic effects, suggesting that the protective effects of education on age-related cognitive impairment risk cannot fully be explained by prenatal factors. A possible explanation as to why a significant protective effect of education on risk of cognitive impairment but not dementia was observed could be that our cognitive impairment measure taps into learned information, which would be strongly related to education.

Of the other covariates, only higher parity and later birth decade were significant for dementia, and increased age and parity for cognitive impairment. The finding that higher parity increases the risk for cognitive dysfunction later in life is in line with previous findings showing an increased risk for low intellectual performance with increased parity [46]. Further, the increased dementia risk with higher birth decade is likely a reflection of an increased sensitivity of the different medical registers over time (the likelihood for dementia to be captured and diagnosed) rather than being due to an increase in dementia incidence. For example, the outpatient register only became part of the NPR in 2001. Further, those born in the most recent birth decade (i.e., 1946 to 1955) would not yet have reached the peak age of onset of dementia and therefore do not show a higher dementia risk. Birth order had no significant effect on the cognitive outcome measures or any of the analyses.

Effect sizes of the within-pair analyses, which are adjusted for shared influences, were of similar magnitude (though not significant) to those from between-pair analyses, suggesting that the increased risk for cognitive dysfunction for individuals who were of smaller birth size is likely not explained by underlying shared etiology, such as genetic factors or shared environmental influences (e.g., prenatal family environment or maternal factors including family SES). These findings, though hampered by a small sample size and low power, could suggest that the smaller twin was exposed to a less optimal intrauterine environment, which manifested itself in the less ideal birth characteristics as well as a worse trajectory throughout the lifetime in terms of cognitive development and cognitive aging. These results are in line with past reports of effects of poor fetal growth (as indicated by adverse birth characteristics) on cognitive development in early childhood based on co-twin control analyses [65]. However, as the HRs and ORs were not significant in the within-pair analyses and confidence intervals were wide, likely due to the small sample sizes, the results should be interpreted with caution.

In light of the past literature, our findings show that small birth size and low fetal growth may increase the risks of age-related cognitive dysfunction late in life even when controlling for familial factors and childhood SES as well as education in adulthood. Further, each additional 100 g BW and additional millimeter HC significantly reduced the risk of dementia and cognitive impairment, respectively, independent of as well as adjusted for GA. Although our findings suggest that the frequently reported protective value of cognitive reserve (as measured by cognitive ability) on cognitive dysfunction may at least to some degree be explained by factors established as early as during fetal development, the increased risk of age-related cognitive dysfunction associated with small birth size and the indicators of fetal growth measured here was also evident in models corrected for childhood SES and education—predictor and proxy of cognitive ability, respectively. It is known that a multitude of factors influence age-related cognitive decline (as well as cognitive reserve), including genetic and neurological factors, immune response, and various environmental factors such as nutrition, physical activity, family environment, physical and mental trauma, and occupation, which are likely to interact with each other throughout the lifetime. It is probable that exposure to a less optimal intrauterine environment will act negatively on several of the factors named above, including general health as well as brain and cognitive reserve, although it is yet to be determined whether the associations of small birth size and low fetal growth with cognitive decline are mediated by any of those factors. Our findings highlight the importance of preventing prenatal growth restriction and closely monitoring the cognitive development of growth-restricted infants. It has been shown that early life catchup growth may be beneficial for the development of those born with adverse birth characteristics [23], and growing evidence about protective factors for dementia emphasizes a life course model with a key role for early life factors [30]. Various reviews converge on similar suggested interventions to increase cognitive reserve: active treatment of hypertension, more childhood education, physical exercise, social engagement, reducing smoking, and preventing and treating depression (e.g., [30,62]).

As touched upon above, there are some limitations to the use of registry-based data. Among these is the lack of sensitivity of dementia diagnoses, as well as the possibility of mistakes in the birth records (e.g., mix-ups of the twin pairs). Given this, it is even more remarkable that such strong associations between birth characteristics and dementia were observed in the registry data and that the findings could be replicated for cognitive impairment, which was not based on medical records but on a cross-sectional cognitive screening study. As a result, though, the cognitive impairment data had no time-to-event information (i.e., date of onset), which would have been desirable, and had to be modeled as ORs rather than HRs. Another potential caveat of registry-based dementia diagnosis is the potential for misdiagnosis, which has been shown to be especially high in those below the age of 65 years [66]. However, as mentioned above, recent findings on dementia diagnoses in the Swedish patient registries have shown a near perfect specificity [61], suggesting very low rates of misdiagnosis. In addition, the mean age of dementia diagnosis in the present study was 74.31 years, and we found a lower risk for dementia in those in the youngest age group (i.e., age 55–65 years), suggesting that the associations we observe are unlikely to be driven by misdiagnoses in young individuals. Further, the present study only explored associations between birth characteristics and all-cause dementia. The observed associations could be driven by a specific dementia subtype, warranting future research to investigate whether associations vary between dementia types. Measures of birth SES and education were included that may not have been ideal as proxies for baseline cognitive ability (or cognitive reserve). A measure of cognitive ability early in life would have been desirable, but such measures were not available in the samples used. However, education is generally considered a reliable proxy of cognitive ability [67], and the fact that the inclusion of education had no effect on the observed associations suggests that there may be little mediation from cognitive ability.

In addition, the dating (exact GA) of pregnancies would likely have been less accurate at the time the birth data were recorded compared to today, which could potentially explain the non-significant associations observed for GA and some GA-corrected variables. Further, perinatal complications, such as asphyxia, have been associated with developmental deficits in both premature and SGA infants [68], which in turn have been shown to predict worse cognitive development. Both asphyxia and LBW are more common in the second-born than in the first-born twin [69–71]. Therefore, additional analyses adjusting for birth order were conducted: these analyses showed little effect of birth order on cognitive dysfunction and little change to the observed associations. This, in combination with the fact that associations in the expected direction were observed across BW, HC, and BL, including HC independent of GA, and that GA (prematurity) had no main effect on the cognitive dysfunction measures, favors the hypothesis that factors associated with fetal growth explain the associations.

Not unexpectedly given the age of the records, there was information missing in the available birth data. In order to obtain less biased and more precise estimations of the parameters of interest, MI was conducted, which allowed us to include those individuals who would have been omitted in a complete case analysis. However, the overall rate of missingness was only 6.2% in the full sample and 9.7% in the subsample, which is generally regarded as inconsequential [72]. We also conducted a complete case analysis (only individuals who had no items missing) as well as analyses with all available data for each model (reported in S1–S9 Tables for comparison). Comparing the models indicates that overall, as expected with this small amount of missingness, the imputation procedure did not have a large effect on our main findings, with overall very similar effect sizes and *p*-values. An assumption of MI methods is that the data are missing at random. The greatest extent of missingness was found for birth SES. Should the data not be missing at random, it is likely that those missing would be at the lower end of the distribution, potentially resulting in residual confounding from birth SES of the effect sizes. Nevertheless, as mentioned above, the distributions of the birth characteristics and covariates in the present sample were in line with those previously reported for twin samples [55]. Finally, there are some potential limitations inherent to twin data, especially in regards to birth characteristics. Twins may generally be more growth restricted in utero compared to singletons (for a detailed review and discussion, see [73]), raising the concern of the generalizability of findings in twins to the general population. However, twins tend not to differ from the general population in morbidity and all-cause mortality across the life span [74,75], suggesting that twinning does not affect long-term health outcomes. Further, research has repeatedly shown that also in other domains such as cognitive ability, personality, and risk for mental disease, twins are similar to the general population (e.g., [76–78]). As such, it is likely that the present findings are also applicable to the general population. The within-pair analyses were unfortunately hampered by small sample size, which is not easily overcome, given the already very large overall sample size.

In conclusion, in this study, we found that smaller birth size (i.e., LBW and small HC adjusted and unadjusted for GA) is associated with a significant risk for late life cognitive dysfunction, largely independent from education level and childhood SES—factors related to cognitive ability. As such, our findings highlight the importance of preventing prenatal growth restriction, closely monitoring the cognitive development of growth-restricted infants, and exploring the potential of reserve-enhancing interventions to possibly protect individuals at higher risk for late life cognitive dysfunction.

## Supporting information

S1 STROBE Checklist STROBE checklist. (PDF)

S1 Table ICD codes used to identify the different types of dementia. (DOCX)

S2 Table Descriptive statistics for covariates. (DOCX)

S3 Table Main analysis results without imputation using full sample: Dementia diagnosis. (DOCX)

S4 Table Main analysis results without imputation using full sample: Cognitive impairment. (DOCX)

S5 Table Main analysis results only with individuals with all covariates: Dementia diagnosis. (DOCX)

S6 Table Main analysis results only with individuals with all covariates: Cognitive impairment. (DOCX)

S7 Table Main analysis results adjusting for within-pair birth order in the imputed datasets: Dementia diagnosis. (DOCX)

S8 Table Main analysis results adjusting for within-pair birth order in the imputed datasets: Cognitive impairment. (DOCX)

S9 Table Co-twin control analysis results adjusted for birth order. (DOCX)

## Abbreviations

BL: birth length
BLGA: birth length adjusted for gestational age
BW: birth weight
BWGA: birth weight adjusted for gestational age
CDR: Cause of Death Register
DZ: dizygotic
GA: gestational age
HC: head circumference
HCGA: head circumference adjusted for gestational age
HR: hazard ratios
LBW: low birth weight
MI: multiple imputation
MZ: monozygotic
NPR: National Patient Register
OR: odds ratio
SBLGA: short birth length for gestational age
SES: socioeconomic status
SGA: small for gestational age
SHCGA: small head circumference for gestational age
STR: Swedish Twin Registry
YOB: year of birth
