# Supplementary material for: Associations between birth characteristics and age-related cognitive impairment and dementia: A registry-based cohort study
Source: PLoS Med. 2018 Jul 18;15(7):e1002609. doi: 10.1371/journal.pmed.1002609 (PMC6051563; doi:10.1371/journal.pmed.1002609)
Supplement: S1 Table — (DOCX) [file pmed.1002609.s002.docx]

**S1 Table.** ICD codes used to identify the different types of dementia.

| Dementia | ICD-7 | ICD-8 | ICD-9 | ICD-10 |
| --- | --- | --- | --- | --- |
| Alzheimer´s disease | 304-305 | 290 | 290A/B | F00 |
|  |  |  | 331A | G30 |
|  |  |  |  |  |
| Vascular dementia |  | 293.0-1 | 290E | F01 |
|  |  |  |  |  |
|  |  |  |  |  |
| Other dementia | 306 |  | 290X/W | F02-03 |
|  |  |  | 294B | G311 |
|  |  |  | 331B/C/X | G318A |
|  |  |  |  | F051 |
