# Supplementary material for: Associations between birth characteristics and age-related cognitive impairment and dementia: A registry-based cohort study
Source: PLoS Med. 2018 Jul 18;15(7):e1002609. doi: 10.1371/journal.pmed.1002609 (PMC6051563; doi:10.1371/journal.pmed.1002609)
Supplement: S2 Table — (DOCX) [file pmed.1002609.s003.docx]

**S2 Table.** Descriptive statistics for covariates.

|  | **Dementia Sample** | | **Cog. Imp. Sample** |  |
| --- | --- | --- | --- | --- |
|  | **Mean (SD) or Count (%)** | **Range** | **Mean (SD) or Count (%)** | **Range** |
| Mothers age | 30.0 (5.7) | 14 – 47 | 30.7 | 15 - 46 |
| Year of birth | 1946 (8.8) | 1926 – 1960 | 1930 (2.6) | 1926 - 1935 |
| Parity | 1.5 (1.7) | 0 – 15 | 2.0 | 0 – 15 |
| Education years | 11.0 (3.1) | 0 – 20 | 9.29 (3.2) | 0 - 20 |
| High education | 22,804 (78.8) | -- | 2043 (52.7) | -- |
| Low education | 6130 (21.2) | -- | 1836 (47.3) | -- |
| Birth SES – high | 2407 (9.4) | -- | 160 (9.0) | -- |
| Birth SES – middle | 13,571 (53.1) | -- | 881 (49.5) | -- |
| Birth SES – low | 9572 (37.5) | -- | 740 (41.6) | -- |

**Note.** SES, socio-economic status.
