# Supplementary material for: Associations between birth characteristics and age-related cognitive impairment and dementia: A registry-based cohort study
Source: PLoS Med. 2018 Jul 18;15(7):e1002609. doi: 10.1371/journal.pmed.1002609 (PMC6051563; doi:10.1371/journal.pmed.1002609)
Supplement: S3 Table — (DOCX) [file pmed.1002609.s004.docx]

| **Variable** | **Model 1** | | | **Model 2** | | | **Model 3** | | | **Model 4** | | |
| --- | --- | --- | --- | --- | --- | --- | --- | --- | --- | --- | --- | --- |
|  | **N** | **HR (95% CI)** | ***p*** | **N** | **HR (95% CI)** | ***p*** | **N** | **HR (95% CI)** | ***p*** | **N** | **HR (95% CI)** | ***p*** |
| BW (100g) | 35,191 | **0.98 (0.97-1.00)^#^** | **0.016** | 34,799 | **0.98 (0.97-0.99)** | **0.004** | 25,285 | 0.99 (0.97-1.01) | 0.310 | 21,725 | 0.98 (0.96-1.00) | 0.052 |
| LBW | 35,191 | **1.19 (1.04-1.36)** | **0.011** | 34,799 | **1.23 (1.07-1.41)** | **0.004** | 25,285 | 1.15 (0.96-1.38) | 0.128 | 21,725 | 1.20 (0.98-1.48) | 0.071 |
| BWGA | 33,707 | **0.92 (0.86-0.99)** | **0.024** | 33,376 | **0.91 (0.85-0.98)** | **0.013** | 24,507 | 0.94 (0.85-1.04) | 0.202 | 21,074 | **0.89 (0.80-0.99)** | **0.041** |
| SGA | 33,707 | 1.15 (0.77-1.72) | 0.497 | 33,376 | 1.17 (0.78-1.75) | 0.452 | 24,507 | 1.19 (0.70-1.99) | 0.519 | 21,074 | 1.12 (0.60-2.11) | 0.715 |
| HC (mm) | 34,166 | 1.00 (0.99-1.00) | 0.394 | 33,797 | 1.00 (0.99-1.00) | 0.371 | 24,773 | 1.00 (0.99-1.00) | 0.493 | 21,278 | 1.00 (0.99-1.00) | 0.226 |
| HCGA | 32,754 | 0.99 (0.91-1.07) | 0.724 | 32,433 | 0.99 (0.92-10.7) | 0.834 | 24,015 | 0.98 (0.88-1.08) | 0.675 | 20,644 | 0.96 (0.86-1.08) | 0.518 |
| SHCGA | 32,754 | **1.62 (1.09-2.40)** | **0.016** | 32,433 | **1.63 (1.10-2.41)** | **0.015** | 24,015 | **2.06 (1.31-3.26)** | **0.002** | 20,644 | **2.19 (1.30-3.66)** | **0.003** |
| BL (cm) | 34,979 | 0.98 (0.96-1.01) | 0.137 | 34,595 | 0.98 (0.95-1.00) | 0.083 | 25,198 | 0.98 (0.95-1.02) | 0.328 | 21,645 | 0.97 (0.94-1.01) | 0.134 |
| BLGA | 33,521 | 0.97 (0.90-1.04) | 0.325 | 33,195 | 0.96 (0.90-1.03) | 0.309 | 24,431 | 0.95 (0.87-1.05) | 0.332 | 21,005 | 0.93 (0.84-1.04) | 0.196 |
| SBLGA | 33,521 | 1.41 (0.99-2.01) | 0.061 | 33,195 | 1.41 (0.98-2.01) | 0.062 | 24,431 | 1.53 (0.97-2.40) | 0.066 | 21,005 | **1.66 (1.01-2.73)** | **0.044** |
| GA (week) | 33,707 | 0.99 (0.96-1.02) | 0.562 | 33,376 | 0.99 (0.96-1.02) | 0.440 | 24,507 | 0.99 (0.96-1.03) | 0.708 | 21,074 | 0.98 (0.94-1.02) | 0.301 |
| Preterm | 33,707 | 0.96 (0.81-1.13) | 0.607 | 33,376 | 0.98 (0.83-1.15) | 0.810 | 24,507 | 0.98 (0.80-1.21) | 0.858 | 21,074 | 0.99 (0.79-1.25) | 0.953 |

**S3 Table.** Hazard ratios for dementia diagnosis based on survival analyses in relation to birth characteristics. Estimates are shown unadjusted (model 1) and adjusted for YOB (in 10-year intervals), sex, and age of mother and parity (model 2); YOB, sex, and age of mother and parity, and birth SES (model 3); and YOB, sex, and age of mother and parity, birth SES, and education level (model 4). Variables and covariates with missing cases were included resulting in different N’s per model. Significant estimates are in bold.

**Note.** BL, birth length; BLGA, birth length adjusted for gestational age; BW, birth weight; BWGA, birth weight adjusted for gestational age; GA, gestational age; HC, head circumference; HCGA, head circumference adjusted for gestational age; HR, hazard ratio, LBW, low birth weight; SBLGA, small birth length for gestational age; SES, socioeconomic status; SGA, small for gestational age; SHCGA, small heard circumference for gestational age; YOB = year of birth.

^#^ upper CIs of 1.00 for significant estimates are rounded (i.e. below 1.00 but higher than 0.995).
