# Supplementary material for: Associations between birth characteristics and age-related cognitive impairment and dementia: A registry-based cohort study
Source: PLoS Med. 2018 Jul 18;15(7):e1002609. doi: 10.1371/journal.pmed.1002609 (PMC6051563; doi:10.1371/journal.pmed.1002609)
Supplement: S5 Table — (DOCX) [file pmed.1002609.s006.docx]

**S5 Table.** Hazard ratios for dementia diagnosis based on survival analyses in relation to birth characteristics. Estimates are shown unadjusted (model 1) and adjusted for YOB, sex, age of mother and parity (model 2); YOB, sex, age of mother, parity, and birth SES (model 3); and YOB, sex, age of mother, parity, birth SES, and education level (model 4). Only individuals with all covariates were included in the analyses. Significant estimates are in bold.

| **Variable** |  | **Model 1** |  | **Model 2** |  | **Model 3** |  | **Model 4** |  |
| --- | --- | --- | --- | --- | --- | --- | --- | --- | --- |
|  | **N** | **HR (95% CI)** | ***p*** | **HR (95% CI)** | ***p*** | **HR (95% CI)** | ***p*** | **HR (95% CI)** | ***p*** |
| BW (100g) | 21,725 | 0.99 (0.97-1.01) | 0.159 | 0.98 (0.96-1.00)^#^ | 0.050 | 0.98 (0.96-1.00) | 0.051 | 0.98 (0.96-1.00) | 0.053 |
| LBW | 21,725 | 1.15 (0.94-1.40) | 0.167 | 1.20 (0.98-1.47) | 0.070 | 1.20 (0.99-1.47) | 0.071 | 1.20 (0.98-1.47) | 0.072 |
| BWGA | 21,074 | 0.92 (0.83-1.02) | 0.099 | **0.90 (0.81-1.00)^#^** | **0.044** | **0.90 (0.81-1.00)^#^** | **0.045** | **0.90 (0.81-1.00)^#^** | **0.044** |
| SGA | 21,074 | 1.17 (0.66-2.07) | 0.597 | 1.18 (0.66-2.11) | 0.568 | 1.18 (0.66-2.11) | 0.570 | 1.18 (0.66-2.11) | 0.568 |
| HC (mm) | 21,278 | 1.00 (0.99-1.00) | 0.378 | 1.00 (0.99-1.00) | 0.220 | 1.00 (0.99-1.00) | 0.224 | 1.00 (0.99-1.00) | 0.229 |
| HCGA | 20,644 | 0.97 (0.87-1.09) | 0.598 | 0.96 (0.86-1.08) | 0.526 | 0.97 (0.86-1.08) | 0.537 | 0.96 (0.86-1.08) | 0.535 |
| SHCGA | 20,644 | **2.02 (1.21-3.37)** | **0.007** | **2.04 (1.22-3.40)** | **0.007** | **2.03 (1.21-3.40)** | **0.007** | **2.03 (1.21-3.40)** | **0.007** |
| BL (cm) | 21,645 | 0.98 (0.94-1.02) | 0.273 | 0.97 (0.94-1.04) | 0.130 | 0.97 (0.94-1.01) | 0.131 | 0.97 (0.94-1.01) | 0.133 |
| BLGA | 21,005 | 0.95 (0.86-1.05) | 0.288 | 0.94 (0.85-1.04) | 0.201 | 0.94 (0.85-1.04) | 0.205 | 0.94 (0.85-1.04) | 0.204 |
| SBLGA | 21,005 | **1.65 (1.02-2.65)** | **0.040** | **1.65 (1.02-2.66)** | **0.041** | **1.65 (1.02-2.66)** | **0.041** | **1.65 (1.02-2.66)** | **0.042** |
| GA (week) | 21,074 | 0.98 (0.94-1.02) | 0.382 | 0.98 (0.94-1.02) | 0.302 | 0.98 (0.94-1.02) | 0.301 | 0.98 (0.94-1.02) | 0.301 |
| Preterm | 21,074 | 0.98 (0.78-1.22) | 0.830 | 0.99 (0.79-1.25) | 0.947 | 0.99 (0.79-1.25) | 0.955 | 0.99 (0.79-1.25) | 0.956 |

**Note.** BL, birth length; BLGA, birth length adjusted for gestational age; BW, birth weight; BWGA, birth weight adjusted for gestational age; GA, gestational age; HC, head circumference; HCGA, head circumference adjusted for gestational age; HR, hazard ratio; LBW, low birth weight; SBLGA, small birth length for gestational age; SES, socioeconomic status; SGA, small for gestational age; SHCGA, small head circumference for gestational age; YOB, year of birth.

^#^ upper CIs of 1.00 for significant estimates are rounded (i.e. below 1.00 but higher than 0.995).
