# Supplementary material for: Associations between birth characteristics and age-related cognitive impairment and dementia: A registry-based cohort study
Source: PLoS Med. 2018 Jul 18;15(7):e1002609. doi: 10.1371/journal.pmed.1002609 (PMC6051563; doi:10.1371/journal.pmed.1002609)
Supplement: S9 Table — (DOCX) [file pmed.1002609.s010.docx]

**S9 Table.** Results of within-pair analyses in twin-pairs discordant for birth characteristics and dementia or cognitive impairment, respectively, adjusted for familial factors shared within twin pairs and additional adjustment for birth order.

| Variable | MZ pairs only | | | MZ and same-sex DZ pairs | | |
| --- | --- | --- | --- | --- | --- | --- |
| Dementia | N pairs | HR or OR (95% CI) | *p*-value | N pairs | HR or OR (95% CI) | *p*-value |
| Birth weight (z-scores) | 88 | 1.19 (0.69 – 2.03) | 0.532 | 214 | 1.05 (0.76 – 1.45) | 0.755 |
| Head circumference (z-scores) | 69 | 0.98 (0.56 – 1.73) | 0.946 | 159 | 0.98 (0.65 – 1.48) | 0.927 |
| Cognitive Impairment |  |  |  |  |  |  |
| Birth weight (z-scores) | 21 | 0.64 (0.17 – 2.47) | 0.521 | 49 | 0.75 (0.37 – 1.52) | 0.419 |
| Head circumference (z-scores) | 15 | 0.88 (0.26 – 2.91) | 0.828 | 36 | 0.68 (0.31 – 1.51) | 0.348 |

**Note.** DZ, dizygotic; HR, hazard ratio; MZ, monozygotic; OR, odds ratio.
